# The clinical association between coagulation indexes, platelet-related parameters, and bone metastasis of newly diagnosed prostate cancer

**DOI:** 10.1186/s40001-023-01562-0

**Published:** 2023-12-13

**Authors:** Zhiwei Yu, Mingxue Yuan, Guojun Chen

**Affiliations:** 1 Department of Urology, Ji Ning Third People‘s Hospital, No. 99 Jianshexi Road, Yanzhou District, Jining, 272100 Shandong China; 2 Qinghai University Affiliated Hospital, No. 29 Tongren Road, Xining, 810012 Qinghai China; 3 Department of Breast and Thyroid, Ji Ning Third People‘s Hospital, No. 99 Jianshexi Road, Yanzhou District, Jining, 272100 Shandong China

## Abstract

**Background:**

At present, much evidence shows that many cancers have a high risk of thrombosis. Several studies have shown the prognostic value of platelet-related parameters and coagulation indexes in prostate cancer (PCa). However, the association between platelet-related parameters, coagulation indexes and bone metastasis of Pca is unclear.

**Methods:**

A total of 234 pathologically diagnosed patients with Pca were consecutively collected and stratified into the bone metastasis group and non-bone metastasis group according to the results of the bone scan. ROC curve analysis was used to explore the auxiliary predictive value of single and combined parameters for bone metastasis in Pca patients. Univariate and multivariate Logistic regression analyses were used to determine the relationship between platelet-related parameters, coagulation indexes, and bone metastasis of Pca.

**Results:**

Platelet count (PLT), fibrinogen (Fib), prostate-specific antigen (PSA), and D-dimer (DD) levels of the bone metastasis group were significantly higher than the non-bone metastasis group (*P* = 0.010, *P* < 0.001, *P* < 0.001, and *P* < 0.001, respectively). This study confirmed that PLT, PSA, DD and Fib have auxiliary predictive value for prostate cancer bone metastasis. After the combination of PLT, PSA, DD and Fib, the area under the curve, sensitivity and specificity increased significantly. The univariate logistic analysis demonstrated that PLT (OR: 1.008, *P* = 0.011), DD (OR: 2.690, *P* < 0.001), PSA (OR: 1.073, *P* < 0.001), Gleason score (OR: 7.060, *P* < 0.001), and Fib (OR: 2.082, *P* < 0.001) were significantly positively correlated with bone metastasis of Pca. Multivariate analysis showed that PSA (OR: 1.075, *P* < 0.001), DD (OR: 2.152, *P* < 0.001), Gleason score (OR: 2.904, *P* < 0.001), and Fib (OR: 1.706, *P* < 0.001) were independent risk factors for bone metastasis of Pca after adjusting for Age, BMI and other confounding factors.

**Conclusions:**

Higher platelet, D-dimer, prostate-specific antigen, Gleason score, and fibrinogen levels may predict a worse prognosis in patients with Pca. PLT, DD, and Fib, as readily available and relatively inexpensive indicators, help predict bone metastasis of Pca. It is suggested that PLT, DD and Fib may be helpful in the risk stratification of Pca.

## Introduction

Prostate cancer (Pca) is one of the most common cancers in men. The morbidity of Pca varies significantly in different regions of the world. It is estimated that by 2040, the number of new cases will reach 2.3 million, and deaths will reach 740000 [1]. The morbidity and mortality of Pca are increasing year by year in China [2]. Bone metastasis is the leading cause of death in patients with Pca. Bone metastasis can easily lead to substantial economic and psychological pressure on patients. The diagnosis of bone metastasis mainly depends on an expensive radionuclide bone scan. Therefore, it is crucial to explore potential cheap predictors to evaluate the severity of Pca more accurately.

The *α* particles of platelets affect the formation of tumor blood vessels by releasing anti-vascular endothelial growth factor (VEGF) [3].Plenty of evidence has shown that aggressiveness of malignancy was closely associated with platelet-related parameters and coagulation indexes [4, 5]. Fib is an acute-phase protein produced mainly by the liver, and the Fib level increases during malignancy and inflammation.DD is a cleavage product of fibrin, and its levels rise in cancer cases, disseminated intravascular coagulation, and pulmonary embolism [6]. Fib has been closely related to the poor prognosis of some malignancies, such as respiratory, digestive, and urinary system malignancies [7, 8]. At the same time, high levels of DD are risk factors for increased risk of death from breast, gastric and pancreatic cancer [9]. Lin et al. found that plasma D-dimer and fibrinogen can predict worse prognosis in patients with digestive system cancer [10]. As a small piece of cytoplasm exfoliated from megakaryocytes, platelets play a crucial role in coagulation and maintaining hemostasis following the mechanical injury of the vascular [11]. In addition, aggregates formed by fibrin, platelets, and tumor cells can promote the metastatic potential of tumor cells [12, 13]. It has long been confirmed that tumor cells can exploit platelets for survival, arrest, and extravasation from blood vessels to distant organs [11]. At present, some studies have shown that coagulation factors can predict the poor prognosis of renal cell carcinoma and prostate cancer [14, 15].

However, the relationship between platelet-related parameters, coagulation indexes, and bone metastasis of Pca is unclear. Hence, the current study aimed to identify the association between platelet-related parameters, coagulation indexes, and bone metastasis of Pca.

## Materials and methods

### Patient selection

We retrospectively evaluated the data of 234 patients with newly diagnosed Pca at the Qinghai University Affiliated Hospital from June 2016 to June 2022.The selected patients met the following inclusion characteristics: 1. prostate biopsy guided by transrectal ultrasound and pathologically diagnosed as prostate cancer. 2. The medical records are complete. The excluded patients had the following criteria: 1. prostate cancer was diagnosed before admission and had undergone surgical or endocrine therapy; 2. Accompanied by other malignant tumors; 3. Diseases with systemic or local inflammation, cardiovascular disease, blood system, and immune system, which had effects on platelet-related parameters and coagulation indexes. 4. Patients who have taken any drug that may affect platelet-related parameters or coagulation indexes within 3 months. 5. Thrombosis or thromboembolism occurred recently. 5. receiving any anticoagulant therapy.

### Clinical date and measurement of index

Venous blood samples of all selected patients were taken at admission after 8 h fasting. The level of platelet count (PLT), mean platelet volume (MPV), platelet distribution width (PDW), plateletcrit (PCT), Fib, prothrombin time (PT), activated partial thromboplastin time (APTT), and thrombin time (TT) were determined on a Stago auto analyzer. The concentration of DD was measured using a Hitachi 7600D clinical chemistry analyzer.

### Statistical analyses

SPSS26.0 (IBM Corp, Armonk, NY, USA) and GraphPad Prism9.4 (GraphPad Software, La Jolla, CA)software are selected for statistical analysis. Kolmogorov–Smirnov test was used to analyze the normality of Continuous variables. The measurement data that obey the normal distribution are tested by independent samples test and expressed as mean ± standard deviation (SD). The Chi-square statistic test analyzes qualitative variables, and the results are expressed as numbers and percentages. Univariate Logistic regression analysis was used to reveal the relationship between platelet-related parameters, coagulation indexes, and bone metastasis of Pca. Multivariate Logistic regression analysis was used further to identify independent risk factors for bone metastasis of Pca. *Statistical significance* was defined as *P* < 0.05.

## Results

### Baseline characteristics of the study subjects

In the present study, 234 patients were divided into two groups: the bone metastasis group (*n* = 116, 49.57%) and the non-bone metastasis group (*n* = 118, 50.43%). When the data of the two groups were compared, we found that PLT, Fib, PSA, and DD levels of the bone metastasis group were significantly higher than the non-bone metastasis group (*P* = 0.010, *P* < 0.001, and *P* < 0.001, respectively). There was no significant difference in Age, BMI, MPV, PDW, PCT, APTT, PT and TT between the two groups (*P* = 0.068, *P* = 0.172, *P* = 0.371, *P* = 0.236, *P* = 0.355, *P* = 0.251, *P* = 0.163, and *P* = 0.064, respectively). According to the Gleason score, 234 patients were divided into two groups: the ≦7 group and the > 7 group. We found that the bone metastasis rate varied with the Gleason score (Detailed clinical parameters are presented in Tables 1 and 2).

**Table 1:** Baseline characteristics of the study subjects

Parameter	Normal value range	Bone metastasis group (*n* = 116)	Non-bone metastasis group (*n* = 118)	*t* value	*P* value
Age (years)	–	71.87 ± 9.40	69.82 ± 7.62	− 1.833	0.068
BMI (kg/m^2^)	18.5 ~ 23.9	22.06 ± 1.65	22.38 ± 1.90	1.369	0.172
PLT (× 10^9^/L)	125 ~ 350	196.21 ± 46.78	180.78 ± 43.84	− 2.603	0.010
MPV (fL)	6 ~ 13	10.93 ± 1.39	11.08 ± 1.16	0.896	0.371
PDW (fL)	9 ~ 17	13.07 ± 2.98	13.50 ± 2.54	1.187	0.236
PCT (%)	0.108 ~ 0.282	0.19 ± 0.06	0.20 ± 0.04	0.927	0.355
DD (mg/L)	≦ 0.55	3.18 ± 1.53	1.70 ± 0.92	− 8.984	< 0.001
Fib (g/L)	2 ~ 4	3.94 ± 1.22	3.07 ± 0.95	− 6.100	< 0.001
APTT (s)	22 ~ 38	28.55 ± 5.56	27.81 ± 4.12	− 1.150	0.251
PT (s)	10 ~ 14	10.91 ± 1.30	10.70 ± 0.93	− 1.401	0.163
TT (s)	14 ~ 21	18.63 ± 2.50	19.18 ± 1.99	1.863	0.064
PSA (ng/ml)	0 ~ 4	72.41 ± 26.33	40.02 ± 15.84	− 11.422	< 0.001

BMI: Body Mass Index; PLT: platelet count; MPV: mean platelet volume; PDW: platelet distribution width; PCT: plateletcrit; DD: D-dimer; Fib: fibrinogen; APTT: activated partial thromboplastin time; TT: thrombin time; PT: prothrombin time; PSA: prostate-specific antigen

**Table 2:** Baseline characteristics of the study subjects

Parameter	Bone metastasis group (*n* = 116)	Non-bone metastasis group (*n* = 118)	*χ*^*2*^	*P* value
Gleason score				
≦ 7	40 (34.48)	93 (78.81)	46.859	< 0.001
> 7	76 (65.52)	25 (21.19)		

### Predictive value of PLT, DD, PSA, and Fib

**Fig. 1:**
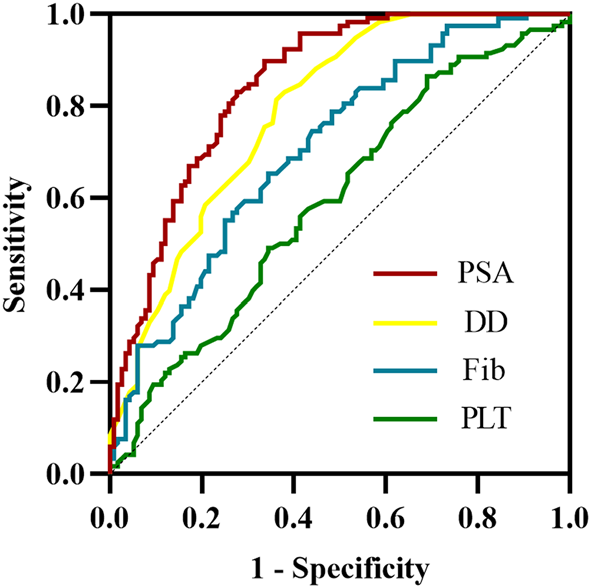
ROC curve of a single parameter

**Fig. 2:**
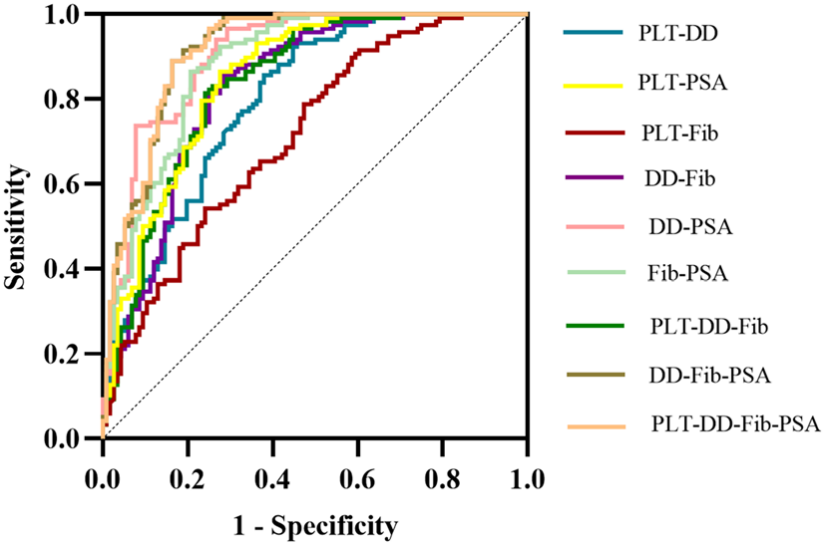
ROC curve of combined parameters

Receiver operating characteristic curve (ROC) analysis results: (1) PLT: the cutoff is 220 × 109/L (the area under the curve is 0.594, the sensitivity is 31.03%, and the specificity is 86.44%). (2) DD: the cutoff is 2.5 mg/L (the area under the curve is 0.790, the sensitivity is 63.79%, the specificity is 81.36%); (3) Fib: the cutoff is 3.4 g/L (the area under the curve is 0.708, the sensitivity is 65.52%, and the specificity is 65.25%). (4) PSA: the cutoff is 59.74 ng/ml (the area under the curve is 0.846, the sensitivity is 66.38%, and the specificity is 89.83%). PLT, DD, PSA, and Fib were combined to predict bone metastasis. The results showed that the area under the curve, sensitivity, and specificity of the combined index were significantly higher than that of the single index. (Detailed data are shown in Table 3 and Figs. 1 and 2).

**Table 3:** Detailed parameters of ROC curve

Parameter	AUC	Cutoff	Sensitivity (%)	Specificity (%)	*P* value
PLT (× 10^9^/L)	0.594	220	31.03	86.44	0.013
DD (mg/L)	0.790	2.5	63.79	81.36	< 0.001
Fib (g/L)	0.708	3.4	65.52	65.25	< 0.001
PSA(ng/ml)	0.846	59.74	66.38	89.83	< 0.001
PLT–DD	0.798	–	62.07	85.59	< 0.001
PLT–Fib	0.714	–	52.59	78.81	< 0.001
PLT–PSA	0.850	–	72.41	86.44	< 0.001
DD–Fib	0.829	–	71.55	85.59	< 0.001
DD–PSA	0.904	–	73.28	94.07	< 0.001
Fib–PSA	0.883	–	79.31	86.44	< 0.001
PLT–DD–Fib	0.837	–	75.86	81.36	< 0.001
DD–Fib–PSA	0.915	–	83.62	88.98	< 0.001
PLT–DD–Fib–PSA	0.915	–	83.62	89.00	< 0.001

PLT: platelet count; DD: D-dimer; Fib: fibrinogen; PLT–DD: the combination of platelet count and D-dimer; DD–Fib: the combination of D-dimer and fibrinogen; PLT–DD–Fib: the combination of platelet count; D-dimer and fibrinogen; PSA: prostate-specific antigen

### Changes of bone metastasis rate in three groups

**Fig. 3:**
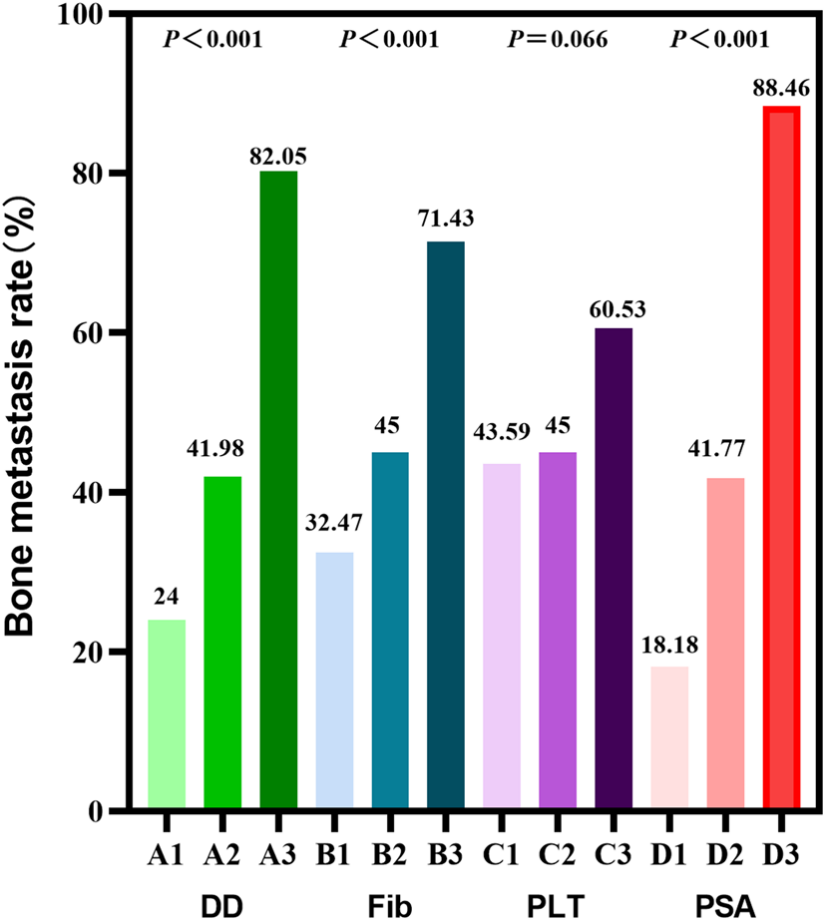
Bone metastasis rate increased with the elevation of DD, PSA, or Fib levels

DD was divided into tertiles according to percentile. The first tertile group: *A*1,  < 1.7 mg/L, 0–33.3th percentile, *n* = 75. The second tertile group: *A*2, 1.7–2.867 mg/L, 33.3–66.7th percentile, *n* = 81. The third tertile group: *A*3,  > 2.867 mg/L, 66.7–100th percentile, *n* = 78. Using the same method, PSA, PLT, and Fib were divided into tertiles. For DD, PSA, and Fib, there were significant differences in bone metastasis rate among the tertiles. Specifically, the bone metastasis rate increased with the elevation of PSA, DD, or Fib levels (*P* < 0.001, *P* < 0.001, *P* < 0.001, respectively). However, there was no significant difference in bone metastasis rate among different levels of PLT (*P* = 0.066) (Detailed data are shown in Fig. 3).

### Relationship between PLT, DD, Fib and bone metastasis of Pca

Univariate analysis demonstrated that PLT (OR: 1.008, *P* = 0.011), DD (OR: 2.690, *P* < 0.001), PSA (OR: 1.073, *P* < 0.001), Gleason score (OR: 7.060, *P* < 0.001) and Fib (OR: 2.082, *P* < 0.001) were significantly correlated with bone metastasis of Pca. Multivariate analysis showed that PSA (OR: 1.075, *P* < 0.001), Gleason score (OR: 2.904, *P* < 0.001), DD (OR: 2.152, *P* < 0.001) and Fib (OR: 1.706, *P* = 0.007)were independent risk factors for bone metastasis of Pca after adjusting for Age, BMI and other confounding factors (Table 4).

**Table 4:** Results of univariate and multivariate logistic regression analysis

Parameter	Univariate mode	*P* value	Multivariate mode	*P* value
	OR (95%CI)		OR (95%CI)	
Age	1.029 (0.998 ~ 1.061)	0.070	–	–
BMI	0.904 (0.781 ~ 1.045)	0.173	–	–
PLT	1.008 (1.002 ~ 1.013)	0.011	1.002 (0.993 ~ 1.011)	0.678
MPV	0.912 (0.745 ~ 1.116)	0.370	–	–
PDW	0.945 (0.861 ~ 1.038)	0.236	–	–
PCT	0.090 (0.001 ~ 14.663)	0.354	–	–
DD	2.690 (2.018 ~ 3.585)	< 0.001	2.152 (1.484 ~ 3.121)	< 0.001
Fib	2.082 (1.589 ~ 2.729)	< 0.001	1.706 (1.154 ~ 2.523)	0.007
APTT	1.031 (0.978 ~ 1.088)	0.251	–	–
PT	1.180 (0.934 ~ 1.489)	0.165	–	–
TT	0.897 (0.799 ~ 1.007)	0.065	–	–
PSA	1.073 (1.053 ~ 1.093)	< 0.001	1.075 (1.050 ~ 1.101)	< 0.001
Gleason score	7.060 (3.940 ~ 12.680)	< 0.001	2.904 (1.190 ~ 7.084)	< 0.001

BMI: Body Mass Index; PLT: platelet count; MPV: mean platelet volume; PDW: platelet distribution width; PCT: plateletcrit; DD: D-dimer; Fib: fibrinogen; APTT: activated partial thromboplastin time; TT: thrombin time; PT: prothrombin time; PSA: prostate-specific antigen

## Discussion

Our study revealed the association between coagulation indexes, platelet-related parameters and bone metastasis of Pca for the first time. In this study, there were significant differences in the levels of PLT, DD, FIB, and PSA between the bone metastasis group and the non-bone metastasis group. It demonstrated the auxiliary predictive value of PLT, DD, PSA, and Fib in bone metastasis. Meanwhile, the combined index will significantly improve the sensitivity and specificity of prediction. PLT, DD, PSA, and Fib, as readily available and relatively inexpensive indicators, help predict bone metastasis of Pca. In addition, with the change of DD, PSA, or Fib level, the bone metastasis rate changed accordingly. In this study, multivariate Logistic regression analysis confirmed that DD, Fib, PSA and Gleason score was independent risk factor for bone metastasis. In summary, higher levels of DD, Fib, PSA and Gleason score may predict a worse prognosis in patients with Pca. We believe that if PLT, DD and FIB are integrated into the existing Pca risk stratification system, it will positively impact the treatment and prognosis of Pca.

It is known that there are many kinds of bioactive molecules in platelet granules. It plays a vital role in the immune escape mechanism of the tumor by expressing different receptors on the surface of platelets [16]. The primary mechanism is that tumor cells are wrapped in thrombus by platelets, thus protecting tumor cells from natural killer cells [11]. Palumbo et al. have corroborated this mechanism; they also found that Fib has a platelet-like effect on the immune escape mechanism of tumor cells [17]. Salih and other researchers have found that an active phenotype on the cell membrane of platelets and tumor cells can secrete large amounts of factors, such as Interferon-γ (IFN-γ) or transforming growth factor-β1 (TGF-β1) [18]. TGF-β1 can decrease the anti-tumor activity of NK cells, mainly by down-regulating the expression of the C-type lectin-like NKG2D receptor [19]. Platelets can protect cancer cells from chemotherapy-induced apoptosis and maintain tumour vessels' integrity [20]. A study shows that elevated platelet levels are associated with poor prognosis in ovarian cancer at first diagnosis and recurrence [21]. A recent study found a positive correlation between platelet count and circulating tumor cells for prostate cancer [22]. In this study, we found that the level of PLT in the bone metastasis group was significantly higher than in the non-bone metastasis group. However, after dividing PLT into tertiles, we found no significant difference in bone metastasis rate among the three groups.

As an essential coagulation factor, fibrinogen promotes tumour cells' progression and invasive potential through various mechanisms. Some researchers believe that tumor cells contain fibrinogen receptors, which can connect fibrinogen molecules to tumor cells, thus improving the endothelial adhesion ability of tumor cell emboli in the blood vessels of target organs [23]. Previous studies have shown that high fibrinogen level is closely related to tumor invasiveness and is an independent risk factor for poor prognosis [24, 25]. For Pca, studies have shown that the level of Fib is positively correlated with the level of prostate-specific antigen, T stage, and Gleason score [4, 26]. The study of Fang-MingWang et al. revealed for the first time the relationship between coagulation parameters, such as fibrinogen and D-dimer and the severity of Pca. They think fibrinogen and D-dimer may be helpful in the risk stratification of prostate cancer [26]. In addition, Fib is an independent prognostic factor in metastatic castration-resistant prostate cancer patients treated with docetaxel [27]. At present, only one study by Xie et al. has explored the relationship between fibrinogen and bone metastasis of Pca. They believe pre-treatment Fib is positively associated with metastatic bone burden in PCa patients [28], which was consistent with our results. We divided fibrinogen into tertiles and found that the rate of bone metastasis increased with the increase of fibrinogen level.

As a biomarker, D-dimer is often used to screen for deep venous thrombosis (DVT), disseminated intravascular coagulation (DIC) and pulmonary embolism (PE) [29]. The elevated D-dimer level indicates coagulation and fibrinolysis system activation. DD, one of the fibrin degradation products, is elevated in many cancer [9, 30, 31]. It is reported that the level of DD is significantly increased in gastric, breast, prostate, and other tumors, and the high level of DD is closely related to tumor progression [32, 33]. A study by Hong et al. showed that DD levels in PCa patients were significantly higher than those in non-prostate cancer patients [34]. Our results suggest that the rate of bone metastasis increases with the increase of DD level.

Our study explored the relationship between platelet-related parameters, coagulation indexes and bone metastasis of Pca. Before us, Xie et al. discussed it and reached a positive conclusion. Nevertheless, they mainly explored the relationship between fibrinogen and the bone metastasis burden of Pca. In this regard, we have improved it and included more parameters. In addition, our study first showed that the bone metastasis rate of pca increased with the increase of DD or Fib levels.

Our study has apparent limitations, and we have not explored the specific mechanism of the relationship between PLT, DD, Fib and bone metastasis. We look forward to the emergence of relevant basic research in the future and exploring specific mechanisms to support our research.

## Conclusions

This study provides new evidence for the correlation between platelet-related parameters, coagulation indexes and poor prognosis of Pca: the higher the level of PLT, DD or Fib, the greater the probability of bone metastasis. This study demonstrated that if PLT, DD and Fib are integrated into the existing Pca risk stratification system, it will positively impact the treatment and prognosis of Pca.

## Data Availability

All data used in this study can be collected from the corresponding authors as required.
